# The HAB1 PP2C is inhibited by ABA-dependent PYL10 interaction

**DOI:** 10.1038/srep10890

**Published:** 2015-06-05

**Authors:** Juan Li, Chaowei Shi, Demeng Sun, Yao He, Chaohua Lai, Pei Lv, Ying Xiong, Longhua Zhang, Fangming Wu, Changlin Tian

**Affiliations:** 1 Hefei National Laboratory for Physical Sciences at the Microscale and School of Life Sciences, University of Science and Technology of China, Hefei, Anhui 230026, P.R. China; 2 High Magnetic Field Laboratory, Chinese Academy of Sciences, Hefei, Anhui 230031, P.R. China

## Abstract

PYL10 is a monomeric abscisic acid (ABA) receptor that inhibits protein phosphatase 2C (PP2C) activity in *Arabidopsis thaliana*. Previous studies reported that the PP2C phosphatase inhibition by PYL10 was ABA-independent. Here, systematic PYL10 biochemical studies demonstrated that PYL10 activity was ABA-dependent, and the previously reported studies was interfered by the presence of BSA in the commercial kit. To investigate dynamic mechanism of how ABA binding to PYL10 induces PP2C phosphatase inhibiting activity, solution NMR relaxation analysis of apo-PYL10 and PYL10/ABA were conducted following backbone resonance assignments. Reduced spectrum density mapping of the backbone relaxation data revealed that PYL10 was more flexible in ABA bound form than apo-PYL10, indicating an increased conformational entropy upon ligand binding. Moreover, to illustrate conformation exchanges of PYL10 upon ABA binding, NMR line shape analysis was performed with increasing concentrations of ABA, and the results indicated that PYL10 backbone conformational changes occur at different time scales.

Abscisic acid (ABA) is an essential phytohormone that contributes to growth, development, and stress responses to drought, salinity or pathogens in plants^1^,^2^. The PYR1/PYL/RCAR family (hereafter referred to as PYLs for simplicity) contains 14 proteins (PYR1 and PYL1-13) in *Arabidopsis thaliana* that have been identified as soluble ABA receptors and therefore play a critical role in ABA signal transduction^3^,^4^. Bioinformatics and crystallographic studies revealed that members of the PYL family are highly homologous in both sequence and structure. PYLs contain a characteristic star-related lipid transfer (START) domain^5^. Upon binding of ABA, PYLs undergo a conformational transition from an open to a closed state via flexible ‘gate’ and ‘latch’ loops, and the resultant ligand-bound conformation can effectively bind to the active site of downstream type 2C protein phosphatases (PP2Cs) to competitively inhibit their phosphatase activity. This subsequently disrupts the PP2C-mediated inhibition of downstream sucrose non-fermenting1-related subfamily 2 (SnRK2) kinases^6^,^7^,^8^,^9^,^10^ that are then activated by autophosphorylation and can stimulate the expression of ABA-responsive genes^11^,^12^,^13^.

Despite the high sequence and structural conservation of PYL proteins, different members display distinct differences in ABA selectivity and sensitivity^14^. Previous biochemical studies suggest that the 14 PYL proteins at least have two mechanism in the inhibition on PP2C: ABA-dependent and ABA-independent. Some dimeric PYLs (such as PYR1, PYL1 and PYL2) are classified as ABA-dependent, since it was reported that they could dissociate and interact with PP2C to inhibit its phosphatase activity upon ABA binding^6^. Some other ABA receptors were recently reported to inhibit PP2C activity in the absence of ABA^15^,^16^. Especially, the monomeric PYLs can form a stronger basal interaction with ABA than do dimeric PYLs^15^. However, whether the monomeric PYLs were really in the mechanism of ABA-independent still need further evidence.

To investigate ABA binding, we recently solved crystal structures of PYL10 in the apo- and ABA-bound forms^17^. These structures were very similar, and both have a closed gate loop (CL2) (Fig. S1B), consistent with the proposed ABA-independent PP2C inhibition of PYL10^16^. However, another crystal structure of the apo- form of PYL10 was reported in an open conformation, with the CL2 region pointing away from the entrance to the ABA binding pocket^16^. Therefore, the CL2 region appears to be highly flexible and able to adopt different conformations, two of which have been captured crystallographically^17^.

Recently, we serendipitously discovered that the PP2C inhibition activity of monomeric PYLs was enhanced by ABA, although this activity was disrupted by BSA in the commercial kinase assay kit. A systematic analysis of dimeric PYL10 and the monomeric mutants was subsequently conducted to verify the ABA dependent activity of monomeric PYLs. Additionally, to exclude conformation effects from crystal lattice packing, solution NMR experiments were performed to analyze the dynamic properties of PYL10 in the absence or presence of ABA. Solution NMR relaxation measurements and consequent dynamic analysis results demonstrated that most residues of PYL10 became more flexible upon ABA binding. We therefore propose that ABA binding to PYL10 leads to elevated conformational entropy (ΔS) and consequently decreased Gibbs free energy (ΔG). Therefore, the ABA binding to PYL10 could enhance the PP2C phosphatase inhibition activities of PYL10.

## Results

### PP2C inhibition activity of PYL10 is enhanced by ABA as well as BSA

**Figure 1:**
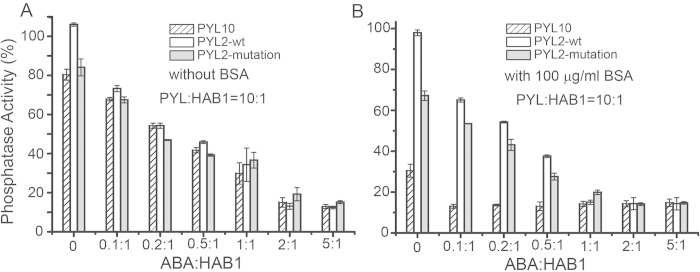
Inhibition of PP2C (HAB1_172-511_) phosphatase by wild type PYL10 and its PYL2-wt (dimer) and PYL2-I88K (monomer) variants. The HAB1_172-511_ illustrated different phosphatase activity in the presence of various concentrations of ABA (ABA : PYL10 : HAB1 = 0:10:1; 0.1:10:1; 0.2:10:1, 0.5:10:1; 1:10:1, 2:10:1, 5:10:1) in the absence (**A**) or presence (**B**) of BSA.

Neighbor-joining phylogenetic tree and biochemical studies have identified at least two classes of ABA receptors; dimeric receptors (PYR1, PYL1-2) and monomeric PYLs (PYL4-10, except PYL7)^15^,^16^. Recently, the monomeric PYL10 was reported to exhibit ABA independent PP2C inhibition activity. During PP2C phosphatase activity experiments in our lab we used home-made buffers instead of those included in commercially available kinase kits (Ser/Thr phosphatase Assay System, Promega, WI, USA), and noticed that the PP2C inhibition activity of PYL10 was disrupted by BSA that is normally included to avoid non-specific interactions (Fig. 1). Different concentrations of ABA were tested in the PP2C (HAB1_172-511_) phosphatase inhibition assay (with PYL10:HAB1 = 10:1), and PP2C phosphatase activity was progressively inhibited (12%–80%) with increasing concentrations of ABA (ABA:PYL10 molar ratio from 0:10 to 5:10), in the absence of BSA (Fig. 1A, hatched bars). This was unexpected and in complete contradiction to the previously proposed ABA-independent activity of PYL10. However, the increase in ABA-dependent PP2C inhibition activity was lost in the presence of 100 μg/ml BSA (Fig. 1B, hatched bars). These results indicated that BSA may compete with ABA binding, especially since BSA is known to bind many different proteins in a non-specific manner^18^.

To verify whether BSA interference was specific to PYL10 or affected all ABA receptors, the dimeric PYL2 and monomeric PYL2 mutants (PYL2-I88K) were subjected to PP2C phosphatase activity inhibition analysis with elevated concentrations of ABA in the absence or presence of 100 μg/ml BSA. The data clearly demonstrated that BSA did not affect the activity of either dimeric PYL2 (Fig. 1A,B, white bars) or monomeric PYL2-I88K (Fig. 1A,B, black bars), suggesting BSA bound specifically to monomeric PYL10, probably leading to the enhanced PP2C phosphatase inhibition activity of PYL10.

**Figure 2:**
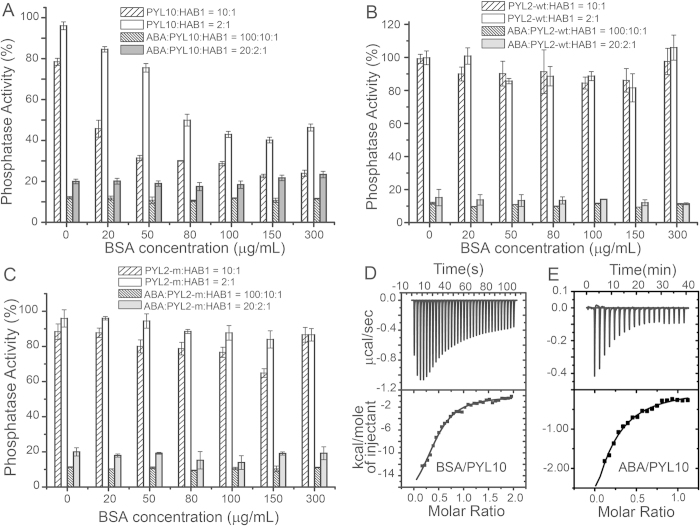
BSA can enhance PP2C phosphatase activity. Inhibition of PP2C (HAB1_172-511_) phosphatase activity by PYL10 (**A**), PYL2-wt (**B**) and PYL2-I88K (**C**) at various concentrations of BSA (0, 20, 50, 80, 100, 150, 300 μg/ml) in the presence or absence of ABA at different ratios of receptor to PP2C (10:1, 2:1). Isothermal titration calorimetry (ITC) analysis of PYL10 with different concentration of BSA (**D**) or ABA (**E**). Dissociation constants were calculated from the ITC data; K_d_ = 3.00 ± 0.27 μM (**D**) for BSA/PYL10, and K_d_ = 66.22 ± 13.86 μM (**E**) for ABA/PYL10. The relative large energy variation of BSA injection relative to ABA injection probably due to the large size of BSA and non-specific binding of BSA to host proteins.

To further investigate the specificity of BSA binding to PYL10, PP2C phosphatase inhibition activity assays were also conducted for PYL10 in the presence of different concentrations of BSA (from 0 to 300 μg/ml) at different PYL10:HAB1 molar ratios (Fig. 2A). In the absence of ABA, PP2C phosphatase activity was inhibited by PYL10 to a greater extent with increasing concentrations of BSA. However in the presence of excessive ABA, PP2C phosphatase activity was strongly suppressed by PYL10 across a broad range of BSA concentrations (Fig. 2A). Therefore, BSA and ABA appear to act competitively in promoting the PP2C phosphatase inhibition activity of PYL10. The ABA-dependent PP2C inhibition activity of dimeric PYL2 or monomeric PYL2-I88K was not obviously affected by BSA (Fig. 1A,B), even at higher BSA concentrations, either in the presence or absence of different concentrations of ABA.

### ABA binding to PYL10 is accompanied by a negative enthalpy change

To further investigate the specificity of ABA and BSA binding to PYL10, isothermal titration calorimetry (ITC) assay was conducted (Fig. S2 & Table S1). The ITC results clearly demonstrated a low affinity of both BSA and ABA for PYL10 (ABA/PYL10: K_d_ = 66.22 ± 13.86 μM; BSA/PYL10: K_d_ = 3.00 ± 0.27 μM; Fig. 2B). Interestingly, both BSA and ABA binding was accompanied by a negative enthalpy change (ABA/PYl10: ΔH = −37.41 ± 22.55 kJ/mol, BSA/PYL10: ΔH = −83.72 ± 5.38 kJ/mol).

The three dimensional structure of apo-PYL10 and ABA-PYL10 show that the residues surrounding the ABA binding pocket include both hydrophilic and hydrophobic residues (Leu79, Leu83, Ile104, Phe155, Leu159 and others; supporting info. Fig. S1). Similarly, the ABA structure also has both hydrophobic and hydrophilic regions. The structure of the ABA/PYL10 protein-ligand complex (PDB 3R6P) shows that PYL10 residue Lys56 stabilizes ABA binding via an interaction with –COOH group of ABA. The PYL10 residues in close proximity to the hydrophobic side of the ABA molecule are hydrophobic (Ile59, Leu79, Leu83, Ile104, Leu113, Tyr116, Phe155, Val156, Leu159 and Ile160), and would therefore provide favorable binding energy. However, the negative enthalpy associated with ABA binding indicates that these hydrophobic interactions do not fully compensate for the lost interactions between solvent molecules and residues in and around the ABA binding pocket. The crystal structure of apo-PYL10 (PDB 3RT2) revealed a network of water molecules that are hydrogen bonded to each other and to a charged and polar side chain, and backbone atoms surrounding the empty PYL10 ABA binding pocket. Binding of ABA would therefore disrupt this hydrogen bonding network, explaining the negative ΔH upon ABA binding to PYL10. We predict that the residues forming the ligand binding pocket may be more flexible following ABA binding since the hydrophobic interactions between ABA and PYL10 may be less energetically favorable than the polar network of interactions in the empty pocket. A site-specific flexibility analysis of PYL10 may provide insight on the molecular mechanism of the ABA-dependent PP2C inhibition activity of PYL10.

### Solution NMR backbone resonance assignment of PYL10 in the absence or presence of ABA

Previous studies demonstrated that the binding of ABA to PYR1, PYL1, or PYL2 correlates with conformational changes at gate and latch loops (CL2 and CL3)^6^,^7^,^8^,^9^,^10^. Unlike other ABA receptors, PYL10 is monomeric rather than dimeric, and the apo-PYL10 structure can adopt both open and closed^17^ conformations. Here, we confirmed that the PP2C phosphatase inhibition activity of PYL10 is ABA-dependent (Figs. 1,2), and a biophysical explanation is highly desirable. It is well known that solution nuclear magnetic resonance (NMR) can provide detailed dynamic information on conformational variations upon ligand binding, and we applied this to investigate monomeric PYL10 in the presence or absence of ABA.

Two-dimensional ^1^H-^15^N hetero-nuclear single quantum correlation spectroscopy (HSQC) was performed on ^15^N labeled PYL10 in aqueous solution, either in the absence or presence of ABA. Interestingly, only around 105 resonances were observed in the spectrum of apo-PYL10, while around 150 resonances were detected in the spectrum of protein-ligand complex (ABA:PYL10 = 4:1; Fig. 3). Many resonances in the two spectra overlap (Supporting Information, Fig. S3), indicating no dramatic conformational differences for residues giving rise to visible HSQC signals. Moreover, it was observed that most resonances in the HSQC spectrum of ABA/PYL10 displayed narrower line-widths than the corresponding resonances in the HSQC spectrum of apo-PYL10 (data not shown).

We took advantage of the increased number of peaks and better resolution of the ABA/PYL10 sample by performing a series of triple resonance NMR experiments to facilitate backbone resonance assignment (^1^H, ^15^N, ^13^CO, ^13^C_α_ and ^13^C_β_) using ^13^C/^15^N double labeled PYL10 in the presence of ABA (ABA:PYL10 = 4:1). From a total of 186 non-proline residues (191 amino acids including five prolines), 144 resonances of PYL10 were assigned. In similar triple resonance NMR experiments on apo-PYL10, only 81 resonance assignments were achieved.

After comparing the two sets of resonance assignments and mapping them to the crystal structure of PYL10 (PDB Number: 3R6P), resonances for many residues could be observed in ABA/PYL10 HSQC spectrum that were not visible in the apo-PYL10 spectrum, including those of α2 (residues 38–47), the 3_10_ helix (residues 51–55), CL1 (residues 56–59), CL2 (residues 79–87), and β7-α3 (residues 140–170). All regions for which resonance assignments could not be made were located at the ABA pocket entrance or the surface at which PP2C binding occurs. This observation indicated that ABA binding may result in conformational mono-dispersion of these regions.

### ABA/PYL10 is more flexible than apo-PYL10 in the nano-second time scale

With the aid of backbone resonance assignments, protein backbone dynamics could be derived from site-specific relaxation measurements. Here, backbone ^15^N longitudinal relaxation (T_1_), transverse relaxation (T_2_) and hetero-nuclear ^1^H-^15^N NOE cross-relaxation data of PYL10 in either apo- or complex forms were obtained using an 850 MHz NMR spectrometer. T_1_, T_2_ or NOE data for ABA/PYL10 (black square) and apo-PYL10 (red circle) are shown in Fig. 4A. The T_1_ longitudinal relaxation values of ABA/PYL10 were similar (N-terminus before α1 and C-terminus region after α3) or larger (other regions) than those of apo-PYL10. Similarly, the average T_2_ transverse relaxation values for ABA/PYL10 were around 25 ms, but only 18 ms for apo-PYL10, and the difference was particularly marked in regions other than the N-terminus (before α1) or C-terminus (after α3). The larger T_2_ values in the ABA/PYL10 data were consistent with the greater number of defined resonances in the HSQC spectrum of ABA/PYL10, suggesting an increased backbone flexibility in the protein-ligand complex. However, the ^1^H-^15^N NOE data of ABA/PYL10 and apo-PYL10 were similar (around 0.75 in major secondary structural regions), indicating a compact conformation of PYL10 in both the absence and presence of ABA.

The global correlation time (τ_c_) can be estimated from the T_2_/T_1_ ratios for residues that satisfy the criteria of NOE ≥0.6 and |T_1_/T_2_ − <T_1_/T_2_>| ≤ SD (standard deviation), since they are unlikely to participate either in slow internal motions or in chemical/conformational exchange processes^19^. The resulting τ_c_ values for apo-PYL10 and ABA/PYL10 were calculated as ~9.22 ns and ~8.12 ns, respectively, which correspond to a molecular weight of about 23 and 21 kDa. Therefore, we could conclude that both apo-PYL10 and ABA-bound PYL10 were monomeric (191 amino acids) in solution, under the experimental solution conditions.

**Figure 3:**
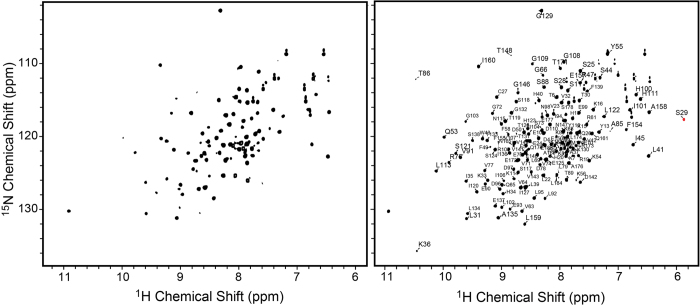
Two dimensional ^1^H-^15^N correlation spectra of PYL10. (**A**) presented the spectra in the absence and (**B**) presence of ABA (ABA:PYL10 = 4:1). Backbone resonances were assigned for the protein-ligand complex.

Given the similar global correlation time of ABA/PYL10 and apo-PYL10, the observed differences in longitudinal or transverse relaxation may reflect the different backbone internal motions of PYL10 in these two states. To quantitatively illustrate backbone internal motions, dynamic information was derived from the measured backbone relaxation data. Since accurate calculation of protein dynamics requires the collection of relaxation data in at least two different NMR fields, spectra were acquired using 600 MHz and 850 MHz instruments. However, low sensitivity and low quality signals in 600 MHz NMR limited the amount of relaxation data acquired. During protein dynamics calculations, model-free motion analysis was based on relaxation data^20^ was attempted initially. However convergence was not reached, possibly due to limited data in the two fields or to lack of resonance assignments and consequent relaxation data from stretch residues in apo-PYL10 (Figs. 3^4^).

**Figure 4:**
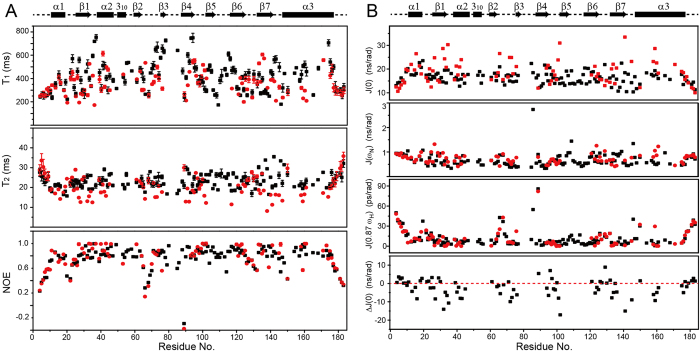
Solution NMR relaxation and dynamic anlaysis of PYL10 in the absence of presence of ABA. (**A**) Residue-specific backbone amide ^15^N T_1_ longitudinal relaxation, T_2_ transverse relaxation and ^1^H-^15^N NOE relaxation measurements. (**B**) Reduced spectral density functions J(0), J(ω_N_), and J(0.87ω_H_) were calculated from the backbone relaxation data in the absence or presence of ABA. ΔJ(0) was calculated from differences between J(0). The secondary structure of PYL10 is shown above.

It is known that reduced spectral density could be more reliably obtained from a single set of relaxation data^21^,^22^. We therefore attempted reduced spectrum density calculations in the hope of generating site-specific internal motion information. Three sets of site-specific spectral density functions were calculated to represent motions in different time scales. Significantly different J(0) values were demonstrated for apo-PYL10 and ABA/PYL10; J(0) values for apo-PYL10 fluctuated between 11 and 33 ns/rad in major secondary structural regions, while J(0) values for ABA/PYL10 averaged 15 ns/rad and fluctuated between 10 and 23 ns/rad. In a site-specific comparison, most J(0) values for ABA/PYL10 were smaller than those of apo-PYL10, as confirmed by the negative J(0) differences (ΔJ(0) = J(0)_ABA/PYL10_ – J(0)_apo-PYL10_; bottom panel, Fig. 4B). J(0) values are reported to be correlated with protein flexibility or general order parameter (S^2^)^23^, and the smaller J(0) values indicated higher protein flexibility in ABA/PYL10. The J(ω_N_) (0.8 ns/rad) and J(0.87ω_H_) (10 ps/rad) values were similar between apo-PYL10 and ABA/PYL10 for the major secondary structural regions (Fig. 4B).

### Structure mapping of the reduced spectral density J(0) values of apo-PYL10 and ABA/PYL10

**Figure 5:**
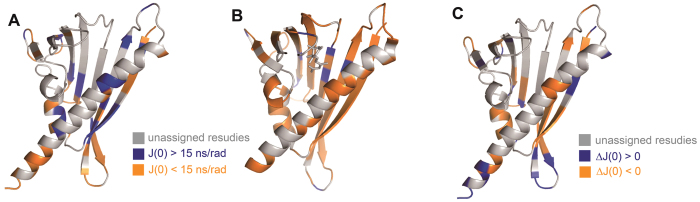
Residue mapping of reduced density functions onto the PYL10 and ABA/PYL10 structures. J(0) values of PYL10 in the absence (**A**) or presence (**B**) of ABA. Differences in J(0) values (ΔJ(0)) in the presence and absence of ABA (**C**) are also shown.

J(0) values represent protein mobility at the nano-second time scale, while lower J(0) values indicates higher flexibility^24^. The site-specific J(0) values determined were mapped to the crystal structures of apo-PYL10 (Fig. 5A) and ABA-PYL10 (Fig. 5B), with J(0) = 15 ns/rad as the threshold. Here, we assumed that J(0) < 15 ns/rad indicated high flexibility, while J(0) > 15 ns/rad indicated slow internal motions. When we compared the distribution of residues with J(0) < 15 ns/rad, together with residue mapping of the J(0) differences (ΔJ(0)), it was obvious that most residues surrounding the ABA binding site in the ABA/PYL10 structure were highly flexible, and relatively few residues (Ala85, Thr86, Glu93, Ile94, Asp96, Asp97,Tyr116, Ser117, Thr119) showed low flexibility. In contrast, most residues surrounding the ligand binding pocket in the apo-PYL10 structure were either highly rigid (blue: J(0) > 15 ns/rad) or a resonance was not assigned, probably due to a slow or medium rate of conformational exchange and consequent broader line-width and low sensitivity. These results strongly indicated that ABA binding increased the conformational flexibility of PYL10, and most regions of the protein were affected. According to the induced-fit theory of protein-protein and protein-ligand interactions, this increased conformational flexibility may lead to a stronger interaction with PP2C and enhanced PP2C phosphatase inhibition by PYL10 following ABA binding.

In terms of thermodynamics, higher flexibility represents increased conformational entropy (ΔS), and ABA/PYL10 has higher conformational entropy than apo-PYL10. Together with the measured decrease in enthalpy (ΔH) upon ABA binding to PYL10 (−37.41 ± 22.55 kJ/mol), the Gibbs free energy (ΔG = ΔH – TΔS) of ABA/PYL10 was lower than apo-PYL10, implying that ABA binding is energetically favorable. Formation of the ABA/PYL10 complex results in increased conformational flexibility of most PYL10 residues, which in turn promotes interaction with PP2C.

Changes in conformational entropy (ΔS) can be calculated from relaxation analysis data from the general order parameters (S^2^) using the formula:
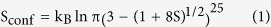


We tried to obtain S^2^ values from backbone amide relaxation data, however the model-free approach failed due to lack of sufficient relaxation data from two or more magnetic fields^25^. Despite the consistency between the J(0) values derived from reduced spectrum density calculations and general order parameter (S^2^)^24^, the quantitative conformational entropy S_conf_ could not be determined without defined analytical derivations from reduced spectrum density functions.

Recent advances in protein backbone relaxation and dynamic analysis have demonstrated variations in flexibility upon ligand binding^26^. Normally, decreased flexibility of proteins upon ligand binding is associated with enthalpy-entropy compensation, consistent with the induced-fit mechanism^26^. However, increased flexibility or conformational entropy can lead to low specificity between the ligand and the protein. Alternatively, increased protein flexibility upon ligand binding might be correlated with cooperativity, resulting in enhanced protein-protein interactions or complex formation^26^,^27^, which is exactly exemplified by ABA promoted PYL10-PP2C interaction. Increased protein flexibility upon ligand binding has been demonstrated before, especially with hydrophobic ligands^25^,^27^. In this study, binding of the hydrophobic ABA resulted in a less rigid form of PYL10 (Fig. 4) that may enhance protein complex formation, consistent with the observed enhanced PYL10-PP2C (HAB1) interactions and consequent increase in PP2C phosphatase inhibition activity (Fig. 1).

### HSQC titration of PYL10 with ABA

**Figure 6:**
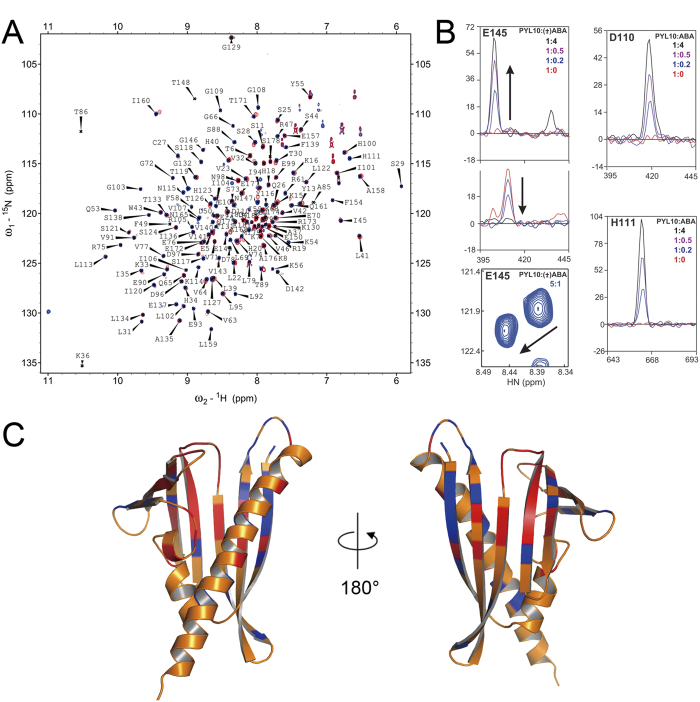
Solution NMR titration of PYL10 with increasing concentrations of ABA (ABA:PYL10 = 0:1, 0.2:1, 0.5:1, 4:1). (**A**) Overlaid two dimensional backbone amide ^1^H-^15^N correlation spectra of PYL10 in the absence and presence of ABA (ABA:PYL10 = 4:1). (**B**) Two different modes of peak changes corresponding to ABA concentration: concerted intensity decrease/increase of neighbouring peaks with different chemical shift values; increasing intensity of constant chemical shift peaks with increasing ABA concentration. (**C**) Residues displaying different peak changes upon ABA titration were mapped to the apo-PYL10 and ABA/PYL10 structures. Peaks displaying concerted intensity increases/decreases at different chemical shifts are colored red; constant chemical shift peaks increasing in intensity with increasing ABA are colored blue; peaks with no obvious intensity or chemical shift value changes are colored orange.

HSQC spectra of apo-PYL10 and ABA/PYL10 shared a similar resonance distribution pattern, indicating similar protein conformations. However, 45 more peaks were present in HSQC spectra of ABA/PYL10 that were absent in apo-PYL10 spectra. A series of HSQC spectra in the presence of gradually increasing concentrations of ABA (ABA:PYL10 = 0:1, 0.2:1, 0.5:1, 4:1) were collected to investigate the protein conformational exchanges involved in ABA binding (Fig. 6). Compared with apo-PYL10 spectra, additional resonances were visible in ABA/PYL0 spectra, even with the addition of small amounts of ABA (ABA/PYL10 = 0.2:1). Upon tracing resonances in the four HSQC spectra, three categories of newly visible peaks were observed: those of constant location and intensity (orange, Fig. 6C); those decreasing in peak intensity with a new peak increasing at a proximal location in the spectra (red, Fig. 6C); those increasing in peak intensity with increasing ABA concentration (blue, Fig. 6C).

For residues not undergoing any obvious changes in chemical shift or intensity during ABA titration (orange, Fig. 6C) are likely to reflect regions of mono-dispersed conformation with relatively high internal motions. These regions are largely confined to the surface of PYL10 and are not likely to be involved in interactions with water molecules in the ligand binding pocket of apo-PYL10 or with ABA in the ABA/PYL10 complex.

Approximately 20 resonances were observed to undergo a drop in peak intensity with increasing concentration of ABA or display an increase in intensity at two constant chemical shift values. Among the peaks displaying a chemical shift, two were observed in intermediate ABA concentrations (Fig. 6B). Most of these resonances were mapped to residues in β1, β7 and CL4 (red, Fig. 6C) that undergo a slow conformational exchange (ms-sec) between ligand-free and ABA-bound forms. β1, β7 and CL4 surround the ligand binding pocket and include many residues that interact with ABA via hydrogen-bonds and other polar interactions. Many of these residues likely also interact with water molecules in the ABA binding pocket of apo-PYL10, consistent with the increased conformational entropy and decreased Gibbs free energy (ΔG) of these regions in ABA/PYL10.

Upon addition of ABA to PYL10, approximately 30 new resonances appeared and maintained constant chemical shift values with increasing ABA (Fig. 6B). These resonances were mapped to residues in CL2, CL3 and consecutive β3, β4, β5, β6 residues around CL2 and CL3 (Fig. 5C). Emerging peaks are strongly indicative that CL2, CL3 and the surrounding β-sheets undergo an intermediate time scale conformation exchange (μs-ms) in apo-PYL10, probably involving several different states. The increasing intensity at a constant chemical shift value for these 30 resonances indicates heterogeneous conformations of apo-PYL10, while faster conformational exchange observed in ABA/PYL10. Upon mapping these residues to the crystal structure of ABA/PYL10, these resonances were found to arise from residues that may interact directly with ABA in the ligand binding pocket. The fast conformation exchange between apo-PYL10 and ABA/PYL10 are probably due to hydrophobic interactions between ABA and hydrophobic residues in the PYL10 ligand binding pocket that replace the network of water molecules in the ligand-free protein. Moreover, the gate, latch and surrounding regions undergo intermediate time scale (μs-ms) conformational exchange or breathing motions in apo-PYL10 that may facilitate ABA entry through the gate region to reach the ligand binding pocket of PYL10.

## Discussion

PYL10 was considered as an ABA-independent PYL since it inhibited the phosphatase activity of PP2C in the absence of ABA in experiments using a commercially available Ser/Thr phosphatase assay kit^16^. In the present study, systematic biochemical analysis of PYL10 using home-made buffers containing ABA revealed an ABA-dependent PYL10 activity in the absence of BSA. We therefore concluded that the previously reported ABA-independent activity may be an artifact due to the presence of BSA in the commercial buffers. Further experiments confirmed that the PP2C phosphatase inhibition activity of PYL10 was indeed ABA-dependent. The PYL family was originally classified into three subfamilies based on phylogenetic analysis; subfamily I (PYL7-10), subfamily II (PYL4-6 and PYL11-13), and subfamily III (PYR1, and PYL1-3)^4^. This classification has subsequently undergone modifications based on biochemical and physiological data. Subfamily 1 contains PYR1 and PYL1-3 that form dimers in solution and are ABA-dependent. Subfamily 2 includes PYL4-10, are monomeric in solution, and are also ABA-dependent. Recent *in vitro* enzymatic studies of PYL13 demonstrated that ABA could not bind to PYL13, and PYL13 could only inhibit phosphatase activity of PP2CA, not other PP2C family members^28^. However, the following studies of PYL13 in *Arabidopsis protoplasts* showed that PYL13 might not be ABA receptor but co-receptor in plants^29^,^30^, indicating that the PYL13 might represent subfamily 3 of PYL family.

With the ABA-dependent activity of PYL10 confirmed, ITC was performed to investigate the specificity of the interactions between PYL10 and ABA or BSA. The observed negative enthalpy of ABA binding indicated an unfavorable interaction between ABA and residues in the ligand pocket of PYL10, which were verified by further comparative dynamic analysis of apo-PYL10 and ABA/PYL10. The results of reduced spectrum density mapping of the backbone relaxation data demonstrated a decrease in J(0) values for most secondary structural regions of ABA/PYL10, indicating elevated flexibility and conformational entropy of following ABA binding. The combination of negative enthalpy (ΔH) and increased conformational entropy (ΔS) of ABA/PYL10 resulted in a decrease in Gibbs energy (ΔG < 0). Therefore, the negative ΔG will lead to more favored ABA-PYL10 interaction, while our systematic enzymatic studies have demonstrated that the binding of ABA to PYL10 could enhance PP2C phosphatase inhibition activity of PYL10 (Fig. 2). Based on these observations, we hypothesized that the increased flexibility of residues in the ABA/PYL10 complex might enhance interactions between PYL10 and PP2C, leading to an increase in PP2C phosphatase inhibition by PYL10. Comparative dynamic analysis of apo-PYL10 and ABA/PYL10 was expanded using HSQC spectrum titration of PYL10 with increasing ABA concentrations, and three categories of peaks were observed. These results provided further evidence that inhibition of PP2C phosphatase activity by PYL10 was modulated by ABA through increased backbone flexibility and conformational entropy. This novel regulatory mechanism may be conserved in other ABA receptors or other physiologically important protein-ligand complexes.

## Methods

### Over-expression and purification of PYL10

The detailed procedure of cloning, over-expression, and purification of PYL10 was performed as previously described^17^. The expression and purification protocol for PYL2 was similar to that of PYL10. The HAB1_172-511_ gene from *Arabidopsis thaliana* was cloned into the pET-21b(+) expression vector (Novagen) and expressed in *Escherichia coli* strain BL21(DE3) (Stratagene) at 25 °C for 18 h. The protein was purified using a Ni-NTA affinity column (QIAGEN) in buffer containing 50 mM Tris-HCl, 500 mM NaCl, 5% glycerol, 5.6 mM β-ME, pH 8.0, and further purified by gel filtration chromatography on a Superdex 200 10/300 GL column (GE healthcare) with buffer containing 50 mM Tris-HCl, pH 8.0, 500 mM NaCl, 5% glycerol and 2 mM DTT on an ÄKTA system Fractions containing protein were concentrated using an Amicon Ultra filter (10,000 MWCO, Millipore).

### Inhibition of PP2C phosphatase activity by PYL receptors

Phosphatase activity was measured using a Ser/Thr phosphatase assay kit (Promega). Each reaction was performed in a 50 μL reaction volume in buffer containing 50 mM imidazole, 5 mM MgCl_2_, 0.02% β-mercaptoethanol, 0.2 mM EGTA, pH 7.5. 0.4 μM HAB1 was added to each reaction with 4 μM of PYL2-wt, PYL2-I88K or PYL10, 40 μM ABA (Sigma-Aldrich) or water, and various concentrations of BSA (Sigma-Aldrich) as required. Reactions were incubated with the peptide substrate supplied in the kit at room temperature for 30 min, and stopped with 50 μL molybdate dye. After incubation for another 15 min at room temperature, the absorbance at 630 nm was measured. At least three independent experiments were performed for each sample.

### ITC of PYL10 with BSA or ABA

ITC experiments were performed using an iTC200 microcalorimeter (MicroCal) in ITC buffer (25 mM HEPES, 100 mM NaCl, pH 6.5) at 30 °C. ABA or BSA were dissolved in ITC buffer, adjusted to pH 6.5, and used directly in titration experiments. Both protein and ligand solutions were degassed extensively, and their concentrations were determined precisely using a UV/Vis spectrophotometer. A total of 20 injections of ABA (1 mM) or BSA (0.2 mM) were made at 120 s intervals into the calorimeter cell which was completely filled with protein solution (0.185 mM or 0.02 mM). Data were analyzed and plotted using Origin software (MicroCal).

### NMR spectroscopy and backbone chemical shift assignments

Protein samples used in NMR experiments were ^15^N or ^15^N/^13^C labeled and contained 50 mM NaPBS buffer (pH 7.2) in a 90% H_2_O/10% D_2_O mixture or in 99.99% D_2_O. All NMR experiments were performed at 30 °C on a Bruker 850 MHz spectrometer equipped with a triple resonance cryo-probe. TROSY based experiments including HSQC, HNCO, HNCACO, HNCA, HNCOCA, HNCACB, and HNCOCACB were carried out for backbone chemical shift assignments. All NMR data were processed using NMRPipe^31^ and analyzed by NMRView^32^ or Sparky 3 (T. D. Goddard and D. G. Kneller, University of California, San Francisco). TALOS+ 33 was used for secondary structure analysis.

### Chemical shift perturbation

A 1.0 mM solution of ^15^N-labeled PYL10 was titrated with 50 mM ABA solution. Four ^1^H-^15^N HSQC spectra were recorded with PYL10:ABA molar ratios of 1:0, 1:0.2, 1:0.5 and 1:4. Spectra were collected on a Bruker 850 MHz NMR spectrometer equipped with a triple resonance cryo-probe. Data processing and analysis were implemented as above.

### Backbone ^15^N relaxation measurements and reduced spectral density mapping

2D ^15^N-^1^H HSQC pulse sequences were used for determination of T_1_ and T_2_ relaxation parameters, and heteronuclear ^1^H-^15^N NOEs. Delay values used for T_1_ and T_2_ experiments were 0.05, 0.1, 0.2, 0.4, 0.8 and 1 s, and 5.5, 11, 16.5, 22, 27.5, 33 and 44 ms, respectively. Heteronuclear ^1^H-^15^N NOE values were determined from peak ratios between presaturated and unsaturated spectra. Reduced spectral density mapping^21^,^22^ was used to analyze the relaxation data. The spectral density function of each individual ^1^H-^15^N amide vector is closely related to its dynamic behavior, and relaxation rates in a two-spin system can be expressed as linear combinations of J(ω) (spectral density function) values at three frequencies: 0, ω_N_ and (0.87ω_H_)^21^,^24^. It is also possible to map the spectral density function using only classical relaxation data: T_1_, T_2_ and ^1^H-^15^N NOEs. The values of J(0), J(ω_N_) and J(0.87ω_H_) were derived from the following four equations:
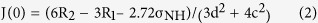

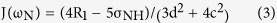

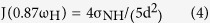

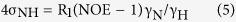
where d = (*μ*_0_*h*γ_N_γ_H_ / (8π^2^)) / (r^3^_NH_), c = ω_N_Δσ / 3^1/2^, *μ*_0_ is the permeability of the vacuum, *h* is the Plank’s constant; γ_N_ and γ_H_ correspond to the gyromagnetic ratios of ^1^H and ^15^N nuclei, respectively; r_NH_ = 1.02 Å and is the average N-H bond length; Δσ is the chemical shift anisotropy for ^15^N nuclei with a value of ~160 ppm^34^.

## Additional Information

**How to cite this article**: Li, J. *et al.* The HAB1 PP2C is inhibited by ABA-dependent PYL10 interaction. *Sci. Rep.* **5**, 10890; doi: 10.1038/srep10890 (2015).

## Supplementary Material

Supplementary Information
