# Supplementary material for: The HAB1 PP2C is inhibited by ABA-dependent PYL10 interaction
Source: Sci Rep. 2015 Jun 5;5:10890. doi: 10.1038/srep10890 (PMC4456664; doi:10.1038/srep10890)

**The HAB1 PP2C is inhibited by ABA-dependent PYL10 interaction**

*Juan Li^1,*^,Chaowei Shi^1,*^, Demeng Sun^1^, Yao He^1^, Chaohua Lai^1^, Pei Lv^1^, Ying Xiong^1^, Longhua Zhang^1^, Fangming Wu^2,#^, and Changlin Tian^1,2,#^*

^1^Hefei National Laboratory for Physical Sciences at the Microscale and School of Life Sciences, University of Science and Technology of China, Hefei, Anhui 230026, P. R. China

^2^High Magnetic Field Laboratory, Chinese Academy of Sciences, Hefei, Anhui 230031, P. R. China

*These authors contributed equally to this work

^#^ Correspondence and requests for materials should be addressed to F.W. (Email: [fmwu@hmfl.ac.cn](mailto:fmwu@hmfl.ac.cn)) or C.T. (Email: [cltian@ustc.edu.cn](mailto:cltian@ustc.edu.cn))

**Figure S1**. Structure of ABA (A) and specific interactions between ABA and residues in ligand binding pocket of PYL10 and CL2 loop(colored carmine)(B), ABA, Lys56 and the hydrophobic amino acids are colored green, orange and yellow, respectively.


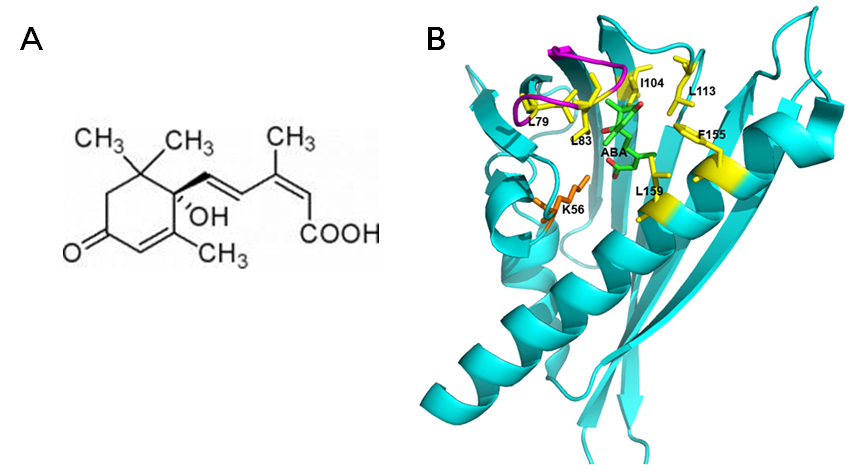


**Figure S2.** The control of titration. (A)BSA into empty cell ; (B)ABA into an empty cell

**
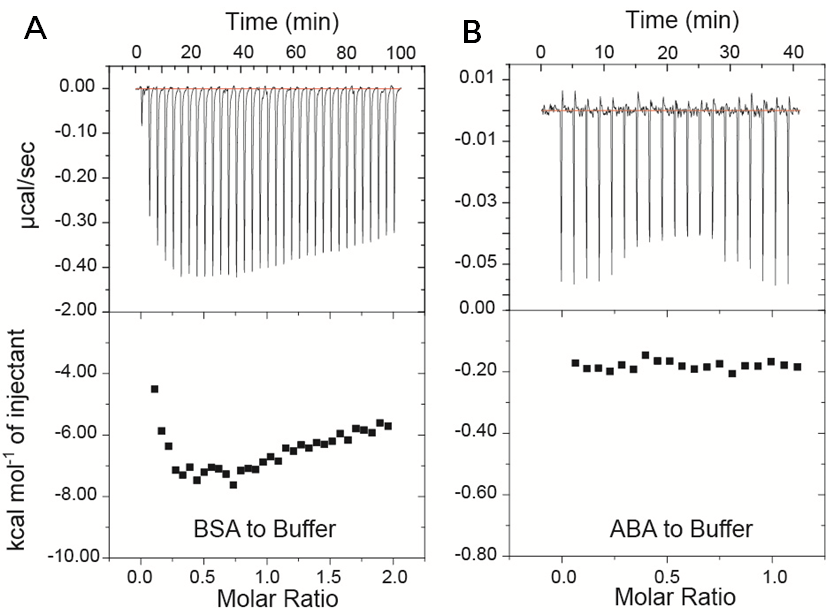
**

**Table S1. The calculated parameters from ITC assay of PYL10 binding with BSA or ABA. (the ITC assay was conducted at 303.15 K or 30** **°C)**

|  | **BSA** | **ABA** |
| --- | --- | --- |
| **Kd(μm)** | **3.00 ± 0.27** | **66.22 ± 13.86** |
| **ΔH (kJ·mol^-1^)** | **-83.72 ± 5.38** | **-37.41 ± 22.55** |
| **ΔS (J·mol^-1^·K^-1^)** | **-174.89** | **29.12** |
| **ΔG (kJ·mol^-1^)** | **-30.70±5.38** | **-46.24±22.55** |

**Figure S3.** ^1^H-^15^N spectra overlay of apo-PYL10 (red) and PYL10/ABA (molar ratio of 1:4) (black).


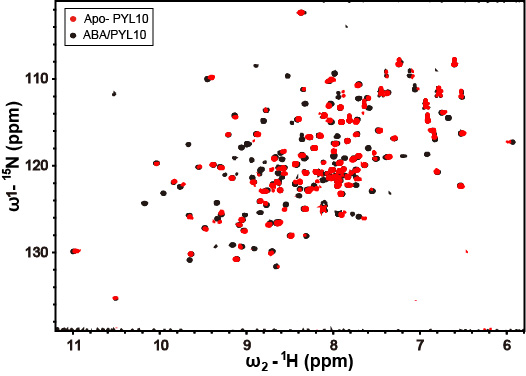

Supplement: Supplementary Information [file srep10890-s1.doc]
